# Multipotent mesenchymal stromal cells as treatment for poor graft function after allogeneic hematopoietic cell transplantation: A multicenter prospective analysis

**DOI:** 10.3389/fimmu.2023.1106464

**Published:** 2023-02-01

**Authors:** Sophie Servais, Frédéric Baron, Chantal Lechanteur, Laurence Seidel, Etienne Baudoux, Alexandra Briquet, Dominik Selleslag, Johan Maertens, Xavier Poire, Wilfried Schroyens, Carlos Graux, Ann De Becker, Pierre Zachee, Aurélie Ory, Julie Herman, Tessa Kerre, Yves Beguin

**Affiliations:** ^1^ Department of Clinical Hematology, University Hospital Center and University of Liège, Liège, Belgium; ^2^ Laboratory of Cell and Gene Therapy, University Hospital Center and University of Liège, Liège, Belgium; ^3^ Department of Biostatistics, SIMÉ, University Hospital Center and University of Liège, Liège, Belgium; ^4^ Department of Clinical Hematology, AZ Sint-Jan Brugge-Oostende AV, Bruges, Belgium; ^5^ Department of Clinical Hematology, University Hospital Leuven, Leuven, Belgium; ^6^ Department of Clinical Hematology, Cliniques Universitaires Saint-Luc, Brussels, Belgium; ^7^ Department of Clinical Hematology, Antwerp University Hospital, Edegem, Belgium; ^8^ Department of Clinical Hematology, Université Catholique de Louvain, University Hospital Center Namur (Godinne), Yvoir, Belgium; ^9^ Department of Clinical Hematology, Vrije Universiteit Brussel, Universitair Ziekenuis Brussel, Brussels, Belgium; ^10^ Department of Clinical Hematology, ZNA Stuivenberg, Antwerp, Belgium; ^11^ Belgian Hematology Society, Brussels, Belgium; ^12^ Department of Clinical Hematology, Ghent University Hospital, Ghent, Belgium

**Keywords:** poor graft function, cytopenia, thrombocytopenia, mesenchymal stromal cells, allogeneic stem cell transplantation

## Abstract

**Introduction:**

Poor graft function (PGF) is a rare but serious complication of allogeneic hematopoietic cell transplantation (alloHCT). Due to their hematopoietic supporting properties and immune regulatory effects, multipotent mesenchymal stromal cells (MSC) could be considered a good candidate to help to restore bone marrow (BM) niches homeostasis and facilitate hematopoiesis after alloHCT.

**Methods:**

We prospectively assessed the efficacy and safety of ex-vivo expanded BM-derived MSC from third-party donor in a series of 30 patients with prolonged severe cytopenia and PGF after alloHCT. This multicenter trial was registered at www.clinicaltrials.gov (#NTC00603330).

**Results:**

Within 90 days post-MSC infusion, 53% (95% CI, 35 – 71%) of patients improved at least one cytopenia (overall response, OR) and 37% (95% CI, 19 - 54%) achieved a complete hematological response (CR: absolute neutrophil count, ANC >0.5 x 10^9^/L, Hb > 80g/L and platelet count > 20 x 10^9^/L with transfusion independence). Corresponding response rates increased to 67% (95% CI, 50 - 84%) OR and 53% (95% CI, 35 - 71%) CR within 180 days after MSC infusion. A significant decrease in red blood cells and platelets transfusion requirement was observed after MSC (median of 30-days transfusion requirement of 0.5 and 0 from d90-120 post-MSC versus 5 and 6.5 before MSC, respectively, p ≤0.001). An increase in ANC was also noted by day +90 and +180, with 3/5 patients with severe neutropenia having recovered an ANC > 1 x 10^9^/L within the 90-120 days after MSC infusion. Overall survival at 1 year post-MSC was 70% (95% CI, 55.4 – 88.5), with all but one of the patients who achieved CR being alive. A single infusion of third-party MSC appeared to be safe, with the exception of one deep vein thrombotic event possibly related to the intervention.

**Discussion:**

In conclusion, a single i.v. infusion of BM-derived MSC from third party donor seemed to improve hematological function after alloHCT, although spontaneous amelioration cannot be excluded. Comparative studies are warranted to confirm these encouraging results.

## 1 Introduction

Allogeneic hematopoietic cell transplantation (alloHCT) offers potential curative treatment for a number of hematological disorders (1). Besides graft rejection and graft failure, there are rare conditions after alloHCT where donor cells engraft but have low hematopoietic functions, resulting in prolonged cytopenias. These situations are referred to as poor graft function (PGF). Multiple definitions have been proposed in the literature for PGF (2–6). Recently, a panel of experts defined PGF as a condition with frequent dependence on red blood cell (RBC) and/or platelet transfusions and/or growth factor support despite donor cell engraftment and in the absence of disease relapse or any other cause (7).

Although rare, PGF is a serious complication after alloHCT and coping with this condition remains a challenge for patients as they may be at increased risk of infections, bleeding events or complications related to iron overload and must undergo an increased number of hospital visits for transfusion support (2, 4, 5, 8). Currently, there is no consensus on how to manage this condition. Most commonly used treatment options include growth factors such as thrombopoietin receptor agonists (TPO-RA), infusion of a donor-derived stem cell boost and second alloHCT (4, 5, 9, 10). However, these options are not always feasible and could be associated with variable efficacity and toxicity. Therefore, there is still room for improvement and development of new therapies to deal with this rare complication.

In transplanted patients, the microenvironment of the bone marrow (BM) hematopoietic stem cell niches can be altered, as a result of damages induced by the hematological malignancy, the conditioning regimen and/or immune mediated graft-versus-host reactions (11, 12). A dysfunctional BM microenvironment may compromise the hematopoietic functions of transplanted stem cells and contribute to the pathogenesis of PGF (4, 5, 13). Mesenchymal stromal cells (MSC) are multipotent progenitor cells that are a major constituent of BM hematopoietic stem cell niches. Compared to patients with good hematopoietic function after alloHCT, it has been shown that BM MSC from patients with PGF exhibited increased intracellular reactive oxygen species, higher levels of apoptosis, accelerated senescence and reduced hematopoietic supportive properties *in vitro* (14). Some studies indicated that MSC play a vital role in supporting HSC self-renewal, differentiation and functions by secreting an array of growth factors and cytokines (15). MSCs can also exert modulatory effects on immune reactions (16).

Co-infusion of MSC with the stem cell graft has been reported to accelerate neutrophil and platelet engraftment after alloHCT (17–21). However, their ability to restore hematopoietic functions when administered after alloHCT in patients with prolonged post-transplant cytopenia has been little explored. Here, we prospectively assessed the safety and efficacy of a single intravenous (i.v.) infusion of ex-vivo expanded BM-derived MSCs from third party donor in patients with PGF after alloHCT.

## 2 Patients and methods

### 2.1 Patient selection

Patients were eligible for this study if they had at least one severe cytopenia (absolute neutrophil count, ANC < 0.5 x 10^9^/L, platelet count < 20 x 10^9^/L, and/or hemoglobin level (Hb) < 80 g/L and reticulocytes < 1%) and/or were dependent on transfusions. Cytopenia(s) had to be present for more than 2 consecutive weeks beyond day + 42 after alloHCT (beyond day +60 in case of cord blood transplantation). Primary PGF was defined as incomplete reconstitution of blood counts since transplantation while secondary PGF was defined as cytopenia appearing after a period of hematological recovery after alloHCT. Patients had to be screened for full-donor chimerism, absence of disease relapse or any other identifiable cause of cytopenia (such as infection, severe acute GVHD, drug-induced myelotoxicity, peripheral destruction) at inclusion. Patients could have received a prior CD34^+^-selected stem cell boost for PGF before study entry, if they were deemed non-responders to this prior treatment by the investigator. Exclusion criteria consisted of HIV seropositivity and active uncontrolled infection.

Eight Belgian centers participated in this study between January 2008 and October 2014. All patients (or their legal guardians) provided written informed consent to enroll in the study. The protocol was approved by the respective ethics review boards of all participating centers and the study was conducted in accordance with the Declaration of Helsinki. This clinical trial was registered at www.clinicaltrials.gov (#NCT00603330).

### 2.2 Mesenchymal stromal cell production and administration

For this study, MSCs were collected from the BM (50 ± 10mL) of 13 third-party healthy volunteer donors (11 men and 2 women) at the CHU of Liège (Liège, Belgium) between 2007 and 2012. Median age of MSC donors was 26 years (range, 20 - 36). Written informed consent was obtained from each donor and the MSC harvest protocol was approved by the institutional ethics review board. MSCs were expanded, cryopreserved and stored in the clinical-grade cell production facility of the Laboratory of Cell and Gene Therapy, CHU and University of Liège (Liège, Belgium). The whole process for donor screening, BM collection, mononuclear cell isolation, MSC expansion, harvesting, cryopreservation, batch selection and thawing procedure, as well as quality control criteria has been described in details elsewhere (22–24). Briefly, MSC were cultured in fetal bovine serum (FBS)-supplemented medium in a normoxic and humidified atmosphere, harvested after 3 passages and cryopreserved in a 10% dimethyl sulfoxide (DMSO)-containing solution.

MSC were administered as a single i.v. infusion at a dose of 1-2 million(s) cells/kg body weight, through a central venous catheter and within 1 hour of thawing. Patients were premedicated with 2 mg/kg methylprednisolone and an anti-histaminic drug.

### 2.3 Hematological response assessment

ANC as well as the numbers of transfused RBC and/or platelet concentrates over 30-day periods were prospectively recorded at day 0 (baseline), +30, +60, +90, +120, +150 and day +180 after MSC infusion.

The primary endpoint was the best hematological response within 90 days after MSC administration (d0-90). Lineage specific response was defined as (1) ANC ≥ 1 x 10^9^/L for neutropenia and (2) Hb > 80g/L and platelet count > 20 x 10^9^/L with no need for transfusion over a 30-day period for anemia and thrombocytopenia respectively. A complete response (CR) was defined as trilineage response, a partial response (PR) as response in at least 1 lineage but with persistence of 1 or 2 cytopenia(s) and nonresponse (NoR) as no improvement of any of the cytopenias. For patients with monolineage cytopenia, only CR and NoR were applicable. Patients were considered to achieve an overall response (OR) if they obtained either CR or PR. In case of death, relapse of the hematological disease or a second transplant before day + 90 after MSC, OR was recorded as the best hematological response before the event (data censored afterwards).

The best hematological responses within 60 days and 180 days after MSC infusion (d0-180) were also analysed.

### 2.4 Other clinical outcomes

Other clinical outcomes included overall survival (OS) and disease relapse within 1 year of MSC infusion. Primary cause of death was defined according to the Copelan hierarchical algorithm (25). Acute and chronic GVHD were monitored and graded according to established criteria at study initiation (26, 27). Serious infectious events (28) were registered within 1 year of MSC infusion. Safety was also carefully monitored.

### 2.5 Statistical analysis

For descriptive statistics, results were expressed as numbers and proportions (%) for qualitative variables and response rates and as medians and ranges for quantitative variables. For univariate analyses, variables were analysed using Wilcoxon rank sum test or logistic regression. Graft CD34+ cell dose and time between alloHCT and MSC infusion were log-transformed to normalize their distributions. A multivariate logistic regression with stepwise selection was applied to identify baseline variables associated with ORd0-90 and CRd0-90. Overall survival (OS) was estimated by Kaplan-Meier curve. The cumulative incidence of relapse was estimated with death and second alloHCT as competing risks and the cumulative incidence of first serious infection with death, second alloHCT and relapse as competing risks. Comparisons of survival between subgroups were performed by the log-rank test. Landmark analyses at day + 90 were performed to compare survival between responders (ORd0-90 or CRd0-90) and non-responders to MSC therapy. Statistical analyses were performed using GraphPad Prism (GraphPad Software, San Diego, CA) and SAS version 9.4 (SAS Institute, Cary, NC, USA). Statistical significance was set at a level of p < 0.05.

## 3 Results

### 3.1 Patients

Thirty patients met eligibility criteria for this study. Patient characteristics are summarized in **Table 1**. The median age at MSC infusion was 51 years (range 11-70; 1 child and 29 adults). All patients had been transplanted for hematological malignancies, the majority of them after a reduced intensity conditioning regimen and with a peripheral blood stem cell graft. The median dose of transfused CD34^+^ stem cells had been 5.5 x 10^6^/kg recipient’s weight. Seven patients had received a graft from HLA-haploidentical donor and 6 from HLA-mismatched unrelated donor. Recipient/donor ABO major or bidirectional incompatibility was present in 6 cases. Six patients had experienced acute graft-versus-host disease and 6 CMV infection after alloHCT and prior to study entry (all these complications were resolved at the time of inclusion).

**Table 1:** Patients characteristics (n= 30).

Patients		
Age, median (range), years < 18 years, n (%)	51 1	(11 – 70) (0.3)
Gender, male, n (%)	20	(67)
Underlying disease		
Acute myelogenous leukemia, n (%) Myelodysplastic syndrome, n (%) Myelofibrosis (primary or secondary), n (%) Other hematological malignancy*, n (%)	15 5 4 6	(50) (17) (13) (20)
Conditioning regimen		
Myeloablative, n (%) Reduced intensity, n (%)	11 19	(37) (63)
Stem cell source		
Peripheral blood stem cells, n (%) Bone marrow, n (%) Umbilical cord blood, n (%)	26 3 1	(87) (10) (3)
Graft CD34^+^ cell dose, median, (range), x 10^6^/kg^#^	5.5	(1.8 – 13)
Type of donor		
HLA-matched sibling, n (%) HLA-matched unrelated, n (%) HLA-haploidentical related, n (%)^§^ HLA-mismatched unrelated, n (%)^§^	6 11 7 6	(20) (37) (23) (20)
ABO major or bidirectional incompatibility, n (%)	6	(20)
Complications after alloHCT		
Acute GVHD, n (%) CMV infection, n (%)	6 6	(20) (20)
Poor graft function after alloHCT		
3/2/1 cytopenia, n (%)	3/17/10	(10)/(57)/(33)
Anemia, n (%) Thrombocytopenia, n (%) Neutropenia, n (%)	26 22 5	(87) (73) (17)
Primary poor graft function, n (%) Secondary poor graft function, n (%)	22 8	(73) (27)
Prior donor CD34^+^ stem cell boost, n (%)	4	(13)
Delay from alloHCT to MSC infusion, median (range), days	159	(42-595)

AlloHCT refers to allogeneic stem cell transplantation; CMV, cytomegalovirus; GVHD, graft-versus-host disease; MSC, mesenchymal stromal cells.

* Other hematological malignancies included: acute lymphoblastic leukemia (n=3), chronic myeloid leukemia (n=1), Hodgkin lymphoma (n=1) and plasma cell leukemia (n=1).

^#^ CD34 ^+^ cell dose not available for 1 patient (umbilical cord blood transplantation).

^§^ All but 1 patient transplanted with non-HLA-matched donors had been screened for absence of anti-HLA donor-specific antibodies prior to alloHCT.

Three patients (10%) were treated for tri-, 17 (57%) for bi- and 10 (33%) for monolineage cytopenia(s). The majority of patients had severe anemia and/or thrombocytopenia. Only 5 patients had severe isolated or combined neutropenia. Overall, 22 patients had primary PGF and 8 secondary PGF. The median delay between alloHCT and MSC infusion was 159 days (range 42-595). Four patients had received a prior CD34^+^ stem cell boost (median dose 4.53 million CD34^+^ cells/kg) for PGF before study entry, with a median delay between boost and MSC infusion of 104 days (42-393). No patient had received or were on treatment with thrombopoietin receptor agonists before or after MSC therapy.

### 3.2 Hematological recovery

**Figure 1:**
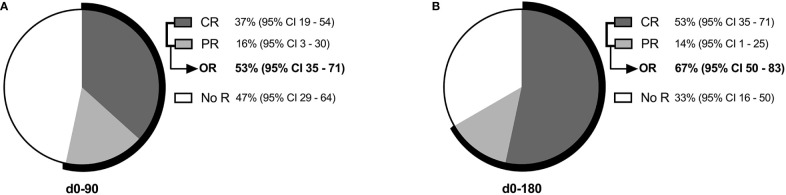
Hematological response **(A)** within 90 days (d0-90) and **(B)** within 180 days (d0-180) of MSC therapy. CR refers to complete response; NoR, no response; OR, overall response (CR + PR); PR, partial response.

Within 90 days after MSC therapy, 16 patients (53%, 95% CI: 35 – 71%) achieved OR and 11 patients (37%, 95% CI, 19 - 54%) achieved CR (**Figure 1A**). Among the 4 patients who had been pre-treated with a CD34^+^ stem cell boost, 1 of them responded to MSC therapy and achieved CRd0-90 (the 3 others had NoRd0-90).

Response rate increased to 67% (20 patients, 95% CI, 50 - 84%) OR and 53% (16 patients, 95% CI, 35 - 71%) CR within 180 days after MSC infusion (**Figure 1B**). All patients who achieved CRd0-90 maintained satisfactory hematological function within the next 3 months (CRd0-180). Two patients with PRd0-90 and 3 with NoRd0-90 converted to CRd0-180.

Considering earlier time-point, eight patients achieved OR (27%, 95% CI: 14-44%) and 3 achieved CR (10%, 95% CI: 3-26%) within 60 days after MSC (**Supplemental Figure S1**).

**Figure 2:**
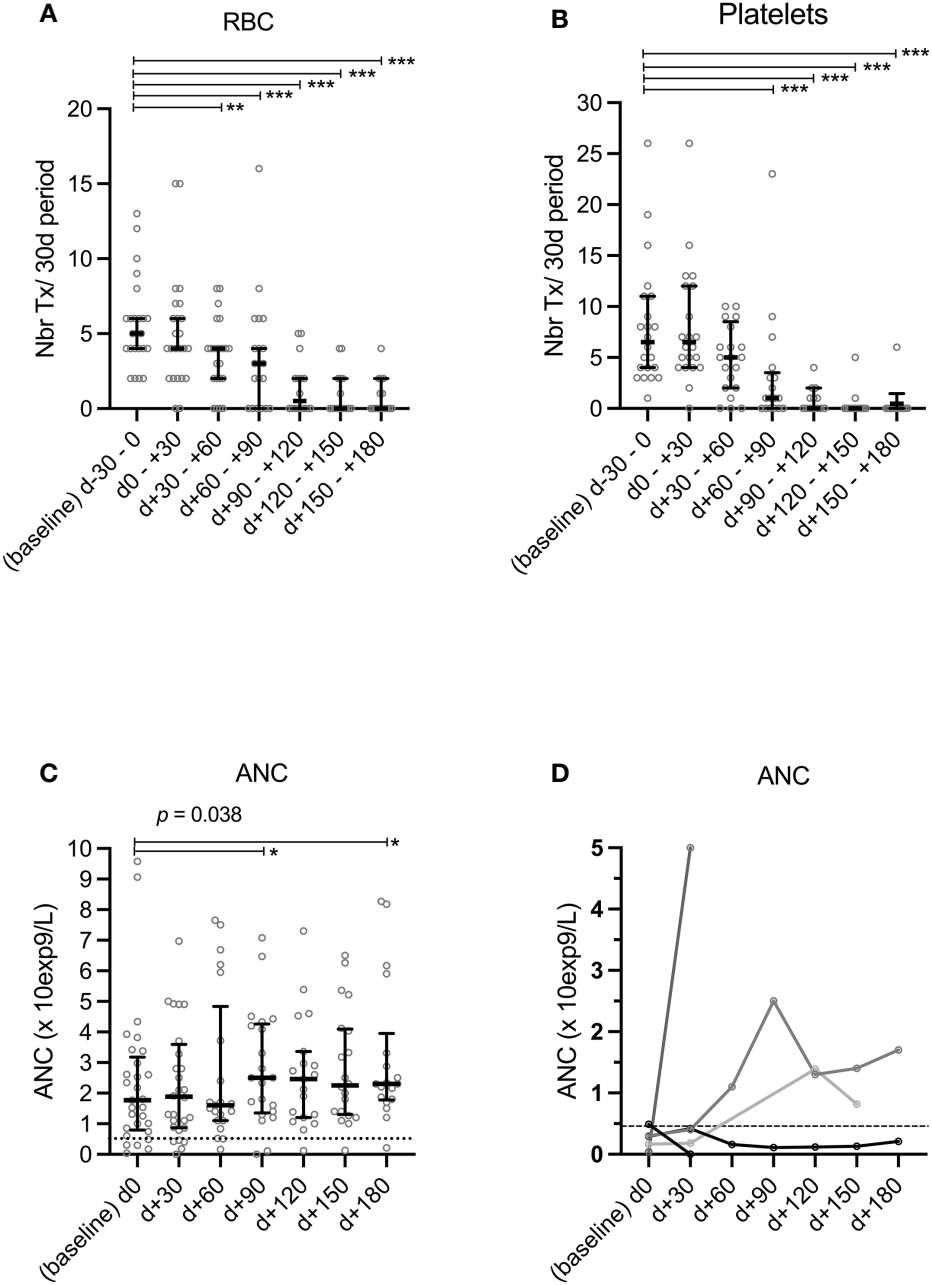
Prospective monitoring of transfusion requirements and ANC after MSC therapy. Numbers of transfused red blood cell (RBC) **(A)** and platelet **(B)** concentrates over 30-day periods and circulating absolute neutrophil counts (ANC) **(C, D)** were prospectively recorded from baseline and up to 180 days after MSC infusion. Data were censored at relapse of hematological malignancy or second transplantation. Among the 5 patients who had severe neutropenia by the time of MSC therapy **(D)**, 1 received a second alloHCT on d+33 after MSC for persistent PGF (NoR at d+30); 1 recovered an ANC > 1x10^9^/L at d+30 then relapsed from the malignant hematological pathology, 2 recovered an ANC > 1x10^9^/L at d90-120 and 1 retained persistent severe neutropenia. *p<0.05, **p<0.01, ***p<0.001 (Wilcoxon rank sum test). ANC refers to absolute neutrophil counts; Nbr Tx, number of transfusions; RBC, red blood cells.

Lineage-specific recovery over time after MSC infusion is depicted in **Figure 2**. Twenty-six and 22 patients suffered from severe anemia and thrombocytopenia, respectively, and were transfusion-dependent before MSC therapy. The median number of transfusions in a 30-day period significantly decreased from d30-60 post-MSC for RBC and from d60-90 for platelet transfusions, and dropped to 0.5 and 0 from d90-120 post-MSC (versus 5 and 6.5 before MSC, respectively, p ≤0.001) (**Figures 2A, B**). An increase in ANC was also observed after MSC therapy in comparison with baseline, which was statistically significant by days + 90 and +180 (**Figure 2C**). Among the 5 patients who had severe neutropenia by the time of MSC therapy, 3 recovered an ANC > 1 x 10exp9/L within 90-120 days after MSC infusion.

We further analyzed associations between several baseline parameters (patient and transplant-related characteristics, history of prior aGVHD and CMV infection, number of cytopenias, primary vs. secondary PGF, prior stem cell boost and delay between alloHCT and MSC infusion) and response to MSC therapy, as assessed by ORd0-90 and CRd0-90, but we did not identify any significant association in multivariate analyses (results of the univariate analyses are illustrated in **Supplemental Table S1**).

### 3.3 Survival

**Figure 3:**
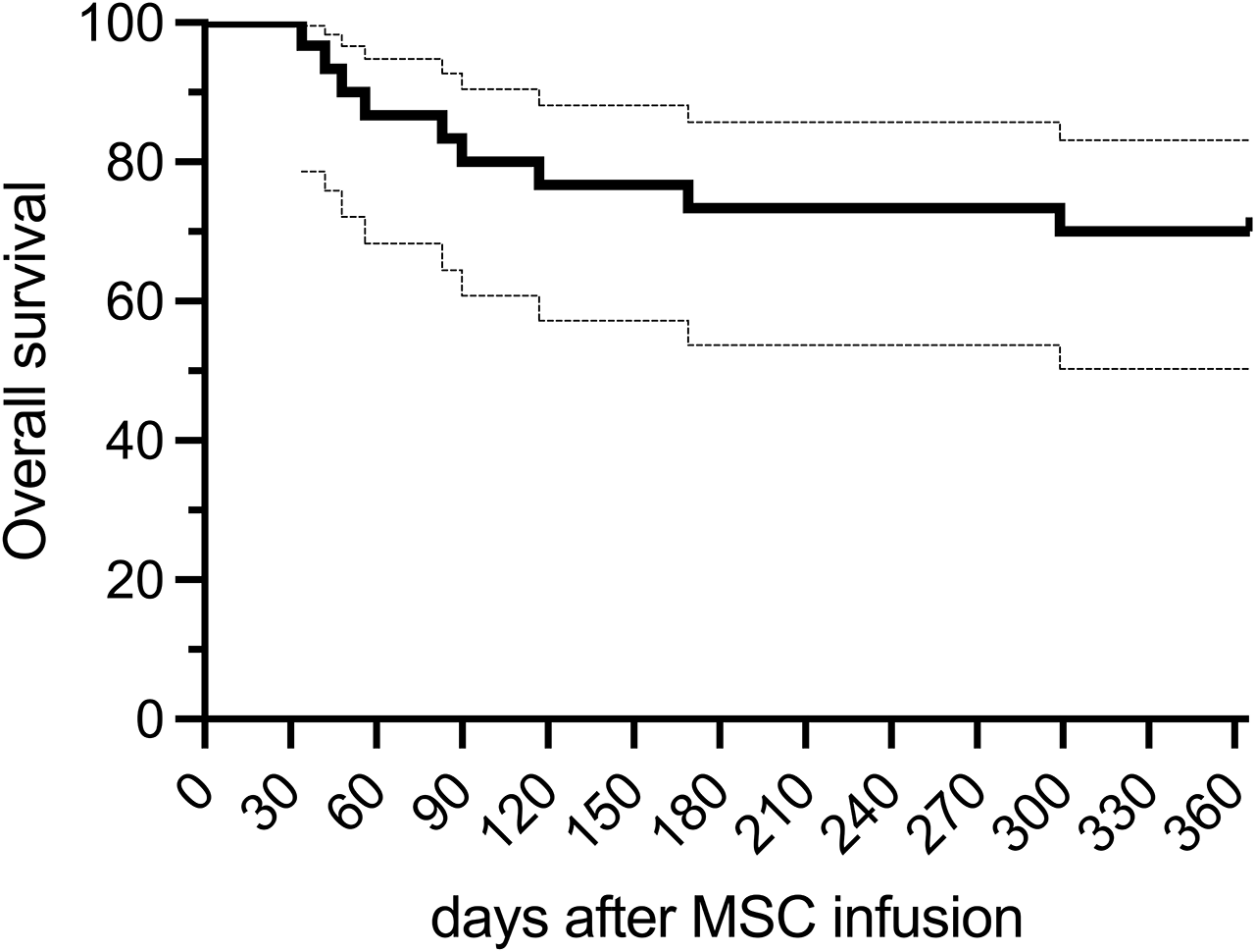
Overall survival (OS) after MSC therapy.

The 1-year OS after MSC therapy was 70% (95% CI, 55.4 – 88.5) (**Figure 3**). Relapse of the hematological malignancy and infections were the leading causes of deaths (**Table 2**). No difference in survival was observed when comparing responders and non-responders to MSC therapy, as assessed by ORd0-90 and CRd0-90 (landmark analysis at day + 90, p= 0.88 and p= 0,61, respectively) (**Supplemental Figure S2**). Of note, 10 of the 11 patients who achieved CRd0-90 were alive at 1 year (1 died because of relapse of hematological malignancy).

**Table 2:** Primary cause of death within 1 year after MSC infusion.

Cause of death	n=
Relapse of hematological malignancy	4
Infection	4*
Acute graft-versus-host disease	1

* 2 pulmonary invasive aspergilloses (day + 42 and + 84 post-MSC; day + 230 and + 158 after alloHCT respectively), 1 staphylococcus aureus pneumonia (day + 169 post-MSC; day + 451 after alloHCT), 1 CMV disease (day + 34 post-MSC; day + 464 after alloHCT). None of these patients had severe neutropenia or active GVHD at MSC infusion or afterwards.

### 3.4 Safety data and other clinical outcomes

No immediate reaction to MSC perfusion was reported. Two deep vein thrombotic (DVT) events were reported as serious adverse events following MSC therapy. The first patient developed seizure due to cerebral sinus thrombosis on day + 8 after MSC infusion (day + 191 after alloHCT for B-cell acute lymphoblastic leukemia). No signs of leukemic relapse, active infection or GVHD were present at the time of thrombosis. The relationship with MSC therapy could not be excluded. The second patient developed deep vein thrombosis of the left arm at day + 199 after MSC therapy. Due to a predisposing factor (central venous implantable device on this side) and the delay after MSC therapy, this event was deemed unrelated to MSC therapy by the investigators. None of these episodes was fatal.

The cumulative incidence of relapse of the hematological malignancy at 1 year after MSC infusion was 20% (95% CI: 7.9–35.9%) (**Supplemental Figure S3**). Median time from MSC infusion to relapse was 92 days (range 28-280 days). No patient developed clinically significant (grade II-IV) acute GVHD or (moderate/severe) chronic GVHD within 1 year after MSC infusion. A total of 30 serious infections (15 of bacterial, 10 of viral and 5 of fungal origin) was recorded during the 1-year follow-up period after MSC therapy. The 1-year cumulative incidence of a first serious infection after initiation of MSC therapy was 60% (95% CI: 39.7-75.4%) and most of them occurred during the first 90 days after MSC therapy (**Supplemental Figure S3**). Four infections were fatal (**Table 2**). No secondary malignancy was observed during the first year after MSC therapy, with the exception of one case of basocellular skin carcinoma (at day + 231 after MSC).

## 4 Discussion

In this multicenter prospective study, we assessed efficacy and safety of a single i.v. infusion of 1-2 millions/kg BM-derived MSCs from third party donors in 30 patients with PGF after alloHCT. MSCs were expanded ex vivo in FBS-supplemented medium in normoxic atmosphere, early passaged and cryopreserved in the setting of an academic clinical-grade cell production facility, thereby ensuring a homogeneous manufacturing process (22, 24). In these conditions, more than half of the patients improved at least one cytopenia and more than one third achieved a complete hematological response to MSC therapy within the 90 days after their infusion. Interestingly, all patients who achieved CRd0-90 maintained a satisfactory hematological function within the next 3 months and all but one of them were alive at 1 year.

These results are consistent with results from previous smaller reports having evaluated BM-derived MSC from third party donors for PGF (29–32). Among them, Liu et al. reported even more encouraging results, with 17 of 20 patients with primary or secondary PGF experiencing hematological response (defined as ANC > 0.5 × 10^9^/L and platelets > 20 × 10^9^/L for 3 consecutive days) to MSC therapy (31). In that study, MSC could be administered for 1 – 3 consecutive courses (at 28-days intervals), based on response to a prior infusion. Whether repeated infusions of MSC may improve the response rate to MSC-based therapy for PGF is unknown. Unfortunately, a direct comparison of our results with this previous study is not possible due to differences in inclusion criteria, definition and timing of response assessment.

Transplantation of donor-derived CD34+-selected stem cell boost and pharmacological therapy with TPO-RA are under investigation as other options for managing prolonged thrombocytopenia and PGF after alloHCT. Two recent systematic reviews have summarized current available evidence utilizing these approaches, respectively (9, 10). They have reported encouraging results in terms of efficacy (with overall responses ranging from 70% to 80%) and acceptable toxicity profile for both of these options, but with the limitations that most of the current evidence is derived from retrospective real-world analyses and case-series, with a potential publication bias toward successful treatment outcomes. Here also, a direct comparison of our results with these studies is difficult due to heterogeneity in inclusion criteria as well as definition of PGF and criteria and timing of response assessment. Future prospective trials, ideally comparative, are needed to determine the efficacy and safety of each of these three options in the management of PGF after alloHCT. Standardization of the definition and timing of evaluation of the hematological response criteria should be recommended in order to homogenize these future clinical trials.

Nevertheless, current evidence of treatment with a stem cell boost or TPO-RA revealed that some patients are refractory to these therapies. Moreover, the option of a stem cell boost could be limited by logistic challenges of donor availability and concerns about risks of GVHD. Whether MSC therapy represents an alternative option for those patients with PGF for whom a CD34^+^-stem cell boost is not feasible or for those who were unresponsive to it and/or to treatment with TPO-RA is an open question. Our cohort included 4 patients with refractory cytopenia after prior therapy with a CD34+ stem cell boost. Among them, one achieved complete hematological recovery within 90 days after MSC therapy. None of our patients had received TPO-RA before or after MSC therapy.

Whether the combination of several approaches (CD34-stem cell boost, TPO-RA, MSC) can lead to beneficial effects in terms of efficacy and toxicity of the procedures needs to be explored in the future. To the best of our knowledge, co-administration of MSC with a CD34+-stem cell boost for PGF has never been explored yet. Recently, Zhu et al. reported their experience of 16 patients with prolonged thrombocytopenia after alloHCT treated with 4-6 weekly administrations of umbilical cord MSC (1×10^6^ cells/kg) in combination with avatrombopag (a second generation TPO-RA) (32). Thirteen of these patients improved their platelet count above 50 x 10^9^/L after a median of 32 days of combined therapy. However, safety and efficacy of this combined approach has to be confirmed in further prospective studies.

The way data was collected and analyzed (over a 30-day period) in our study did not allow us to determine the precise timing of response to MSC therapy. The response rate observed within d0-60 after MSC infusion was approximately two times lower than that observed within d0-90. Does this mean that the hematopoietic supporting effects of MSCs mainly appear during the second month after MSC infusion and carry over to d60-90 or is it the manifestation of a spontaneous hematopoietic recovery over time? It’s impossible to conclude in the absence of a control group. Some other studies reported that neutrophiles and/or platelet recovery after hematopoietic supportive therapies with either MSC or TPO-RA for PGF was indeed generally observed during the second month of treatment (31, 33, 34).

In our study, we could not identify any baseline predictive factor of hematological response to MSC therapy. Our small cohort of patients indicated that MSC appeared to be equally effective for primary and secondary PGF, patients with single and multiple lineage cytopenia(s) and regardless of prior transplantation modalities and prior history of acute GVHD or CMV infection. Nevertheless, our results have to be interpreted with caution, regarding the small number of patients.

Our cohort included 10 patients with monolineage severe cytopenia, and therefore was not strictly limited to patients with at least bilineage cytopenia, as suggested to be the definition of PGF by the European Society of Blood and Marrow Transplantation (EBMT) and some other experts or investigators of recent studies (2–4, 8, 35, 36). However, in our analysis, mono- versus multiple lineage cytopenia did not appear as a factor influencing response to MSC in univariate and multivariate analyses, therefore precluding that this subgroup of patients could have influence the response rate of the overall cohort. Moreover, prolonged severe monolineage cytopenia (i.e. isolated neutropenia or thrombocytopenia) has also been reported to be associated with adverse outcomes after alloHCT (37, 38), could be a challenge to improve and therefore could still represent a real clinical concern. Recently, a panel of experts of the American Society for Transplantation and Cellular Therapy proposed a broader definition for PGF as a situation of frequent dependence on transfusions of RBC and/or platelets and/or growth factor support, without precising the number of cytopenia required to fit the definition (7). Unfortunately, only 5 patients in our cohort had isolated or combined severe neutropenia, thus precluding the possibility to assess the effects of MSC on ANC recovery.

We observed a 1-year OS of 70% after MSC infusion in our cohort, without difference between responders and non-responders to the therapy. This survival rate contrasts with some prior retrospective reports showing very dismal outcome in patients without hematological recovery, with survival as low as 25% and 6% of at 1 and 2 years (2, 8). However, heterogenous outcomes are reported in the literature for patients with PGF (2, 8, 39, 40). Variability in PGF definitions between studies might possibly account for this heterogeneity. The presence or absence of severe neutropenia could likely be a factor influencing the outcomes, with increased nonrelapse mortality being expected in PGF without neutrophil recovery because of the risk of infectious complications. The few numbers of patients with severe neutropenia included in our study might have accounted for the favorable OS of our cohort.

Regarding acute toxicity, MSC i.v. infusion appeared to be safe in our study, with the exception of one deep vein thrombotic event occurring a few days after MSC administration whose relationship to the intervention is not clear. Based on our previously published clinical experience with i.v. infusions of BM-derived MSCs from third-party donors (produced and administered in the same manner as described here), no other deep vein thrombotic event has been observed in more than 200 treated patients for a range of conditions other than PGF (including GVHD after alloHCT, solid organ transplantation, coronavirus disease [COVID-19]) (22–24, 41–45). A recent meta-analysis of prospective randomized controlled trials (RTC) that compared intravascular administration of MSC to controls in various indications in adult patients (55 studies, 2696 patients) did not suggest either an association between MSC treatment and thrombotic/embolic events (46). However, *in vitro* procoagulant effects of MSC on human blood and plasma have been described, although MSC procoagulant phenotype seem to be influenced by multiple factors including the MSC source and manufacturing (passage number, cryopreservation,…) but also patient-related factors (pre-existing inflammation) (47–49). Therefore, although no significant association between MSC and thrombotic events has been detected so far in humans, it is still possible that these events are rare and we encourage researchers to continue to monitor and report them in future MSC studies to confirm the absence of safety signals.

More than half of our patients experienced at least one serious infection within the first year after MSC therapy and four of them died because of infection as primary cause of death. Similarly, Liu et al. reported a high number of infectious events in their cohort of patients with PGF treated with MSC (31). Infections are frequent complications and a leading cause of mortality after alloHCT (50). Susceptibility to infections can also be higher in patients with PGF (as a consequence of neutropenia, iron overload, increased hospitalizations and hospital visits). Whether MSC with immunosuppressive properties could confer an increased risk of serious or fatal infections in this fragile population could not be established from our and Liu’s studies, since there was no comparative control group. Reassuringly, several meta-analyses of RCTs with MSC administered in various clinical settings (not limited to alloHCT) have reported no association between MSC therapy and an increased risk of infections (46, 48).

Limits of our study stem in the small number of patients, inclusion of patients with mono- versus multilineage cytopenia(s), the small numbers of patients with severe neutropenia, and the absence of a control group that could not allow us to apprehend the potential contribution of spontaneous recovery of the hematopoietic function over time.

In conclusion, our study provides encouraging results on the efficacy and safety of MSC-based therapy for PGF and prolonged severe cytopenia after alloHCT. Future studies, ideally comparative, are warranted to confirm them.

## Data availability statement

The raw data supporting the conclusions of this article will be made available by the authors, without undue reservation.

## Ethics statement

The studies involving human participants were reviewed and approved by the respective ethics review boards of all participating centers and by the Comité d’Éthique Hospitalo-Facultaire Universitaire deLiège, Liège, Belgium (central commitee).

## Author contributions

Conception and design: FB, YB; Provision of study material or patients: All authors; Collection and assembly of data: SS, FB, AO, YB; Data analysis and interpretation: SS, LS, YB; Manuscript writing: SS, YB. All authors contributed to the article and approved the submitted version.
