# Supplementary material for: Multipotent mesenchymal stromal cells as treatment for poor graft function after allogeneic hematopoietic cell transplantation: A multicenter prospective analysis
Source: Front Immunol. 2023 Feb 1;14:1106464. doi: 10.3389/fimmu.2023.1106464 (PMC9929549; doi:10.3389/fimmu.2023.1106464)

Supplementary Material

Multipotent mesenchymal stromal cells as treatment for poor graft function after allogeneic hematopoietic cell transplantation: a multicenter prospective analysis

Sophie Servais[1*], Frédéric Baron[1], Chantal Lechanteur[2], Laurence Seidel[3], Etienne Baudoux[2], Alexandra Briquet[2], Dominik Selleslag[4], Johan Maertens[5], Xavier Poire[6], Wilfried Schroyens[7], Carlos Graux[8], Ann De Becker[9], Pierre Zachee[10], Aurélie Ory[11], Julie Herman[11], Tessa Kerre[12] and Yves Beguin[1]

[1]Department of Clinical Hematology, CHU and University of Liège, Liège, Belgium;

[2]Laboratory of Cell and Gene Therapy, CHU and University of Liège, Liège, Belgium;

[3] Department of Biostatistics, SIMÉ, CHU and University of Liège, Liège, Belgium;

[4]Department of Clinical Hematology, AZ Sint-Jan Brugge-Oostende AV, Bruges, Belgium;

[5]Department of Clinical Hematology, University Hospital Leuven, Leuven, Belgium;

[6]Department of Clinical Hematology, Cliniques Universitaires Saint-Luc, Brussels, Belgium

[7]Department of Clinical Hematology, Antwerp University Hospital, Edegem, Belgium;

[8]Department of Clinical Hematology, Université Catholique de Louvain, CHU UCL Namur (Godinne), Yvoir, Belgium;

[9] Department of Clinical Hematology, Vrije Universiteit Brussel (VUB), Universitair Ziekenuis Brussel, Brussels, Belgium;

[10]Department of Clinical Hematology, ZNA Stuivenberg, Antwerp, Belgium;

[11]Data nurse, Belgian Hematology Society, Brussels, Belgium;

[12]Department of Clinical Hematology, Ghent University Hospital, Ghent, Belgium.

*** Correspondence:**
Servais Sophie; Department of Clinical Hematology, CHU and University of Liège, CHU Sart-Tilman, 1 avenue de l'hôpital, 4000 Liège, Belgium.
Tel +32 - 4 – 366 72 01 ; Fax +32 - 4 - 366 88 55
E-mail: s.servais@chuliege.be

Running title: MSC for PGF after alloHCT

Supplemental Table S1 – Associations between baseline parameters and response to MSC therapy as assessed by (A) ORd0-90 and (B) CRd0-90

	(A) ORd0-90			(B) CRd0-90		
Effect	**OR**	**(95% CI)**	***p***	**OR**	**(95% CI)**	***p***
Patient age (years)	1.003	(0.956 - 1.052)	*0.90*	0.987	(0.940 - 1.037)	*0.60*
Patient gender, female vs. male	0.818	(0.179 - 3.744)	*0.80*	0.643	(0.127 - 3.254)	*0.59*
Conditioning regimen, MAC vs. RIC	0.606	(0.136 - 2.705)	*0.51*	0.980	(0.210 - 4.579)	*0.98*
$CD34^+$cells/kg recipient's weight ($x10^6$/kg)	0.314	(0.058 - 1.708)	*0.18*	0.155	(0.022 - 1.101)	***0.062***
Donor type, MUD vs. HLA-id SIB	0.600	(0.076 - 4.761)	*0.86*	0.833	(0.114 - 6.111)	*0.59*
Haplo vs. HLA-id SIB	0.375	(0.039 - 3.605)	.	0.400	(0.040 - 3.955)	.
MMUD vs. HLA-id SIB	0.500	(0.049 - 5.154)	.	0.200	(0.014 - 2.911)	.
ABO major (+/- minor) IC, yes vs. no	2.000	(0.306 -13.06)	*0.47*	0.280	(0.028 - 2.780)	*0.28*
Prior grade II-IV aGVHD, yes vs. no	0.846	(0.141 - 5.070)	*0.85*	0.280	(0.028 - 2.780)	*0.28*
Prior CMV infection, yes vs. no	0.846	(0.141 - 5.070)	*0.85*	0.833	(0.126 - 5.504)	*0.85*
Number of cytopenia, 2-3 vs. 1	0.667	(0.143 - 3.107)	*0.61*	0.222	(0.044 - 1.123)	***0.069***
PGF, secondary vs. primary	0.190	(0.031 - 1.177)	***0.074***	0.171	(0.018 - 1.638)	*0.13*
Time between alloHCT and MSC (days)	0.240	(0.054 - 1.073)	***0.062***	0.488	(0.128 - 1.866)	*0.29*
Prior stem cell boost, yes vs. no	0.244	(0.022 - 2.676)	*0.25*	0.533	(0.049 - 5.862)	*0.61*

AlloHCT refers to allogeneic stem cell transplantation; aGVHD, acute graft-versus-host disease; CMV, cytomegalovirus; Haplo, HLA-haploidentical donor; HLA-id, HLA-identical sibling donor; IC, incompatibility; MAC, myeloablative conditioning regimen; MMUD, HLA-mismatched unrelated donor; MUD, HLA-matched unrelated donor; MSC, mesenchymal stromal cells; PGF, poor graft function; RIC, reduced intensity conditioning regimen.

Supplemental Figure S1 - Hematological response within 60 days (d0-60) of MSC therapy.

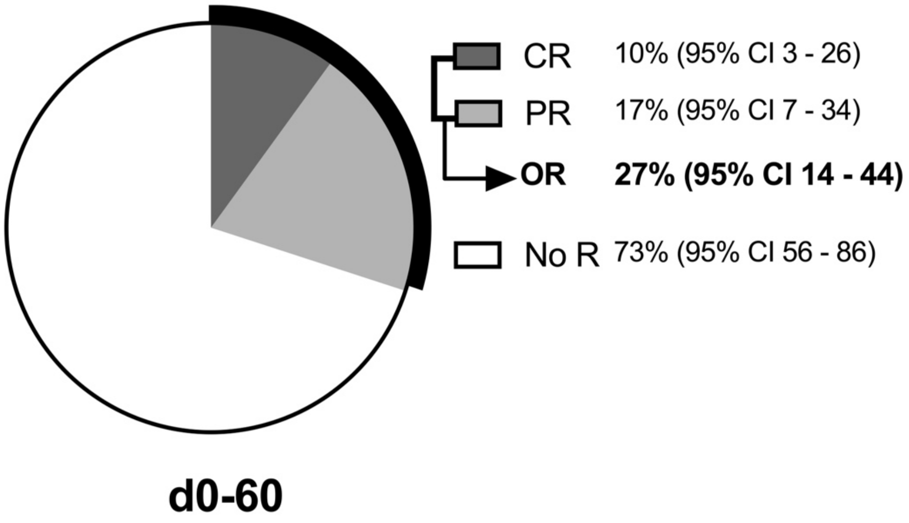

CR refers to complete response; NoR, no response; OR, overall response (CR + PR); PR, partial response

Supplemental Figure S2 – Comparison of overall survival (OS) between responders and non-responders to MSC therapy, as assessed by (A) ORd0-90 and (B) CRd0-90 (Landmark analyses at day + 90).

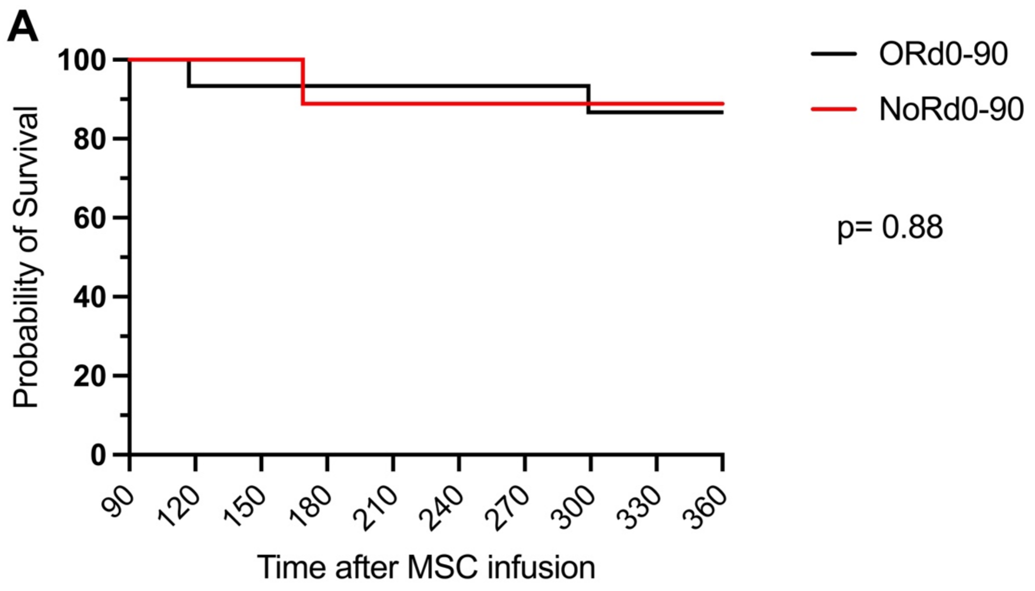

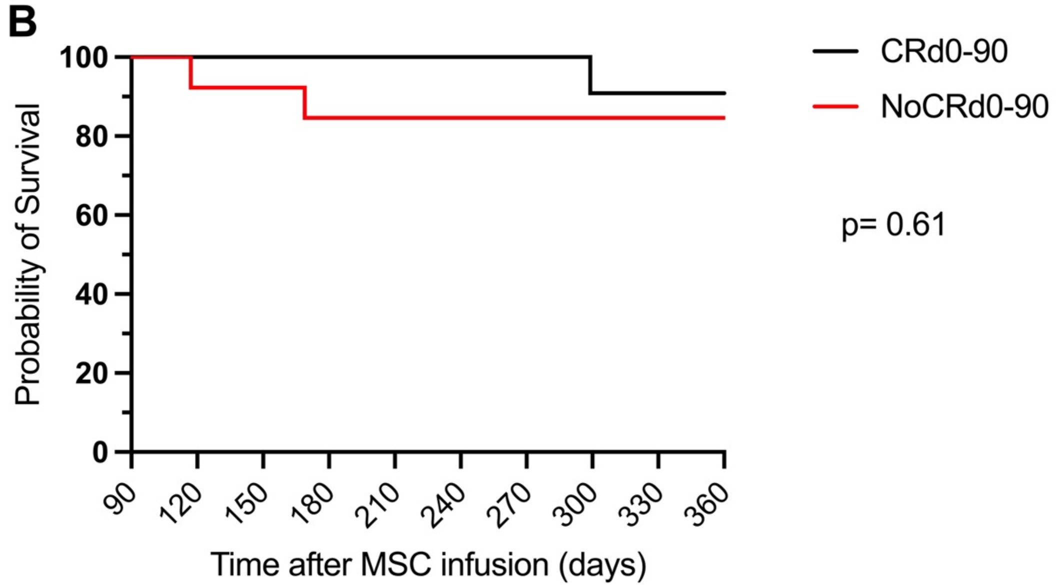

Supplemental Figure S3 - Cumulative incidences of (A) relapse of the hematological malignancy and (B) first infectious event after MSC therapy

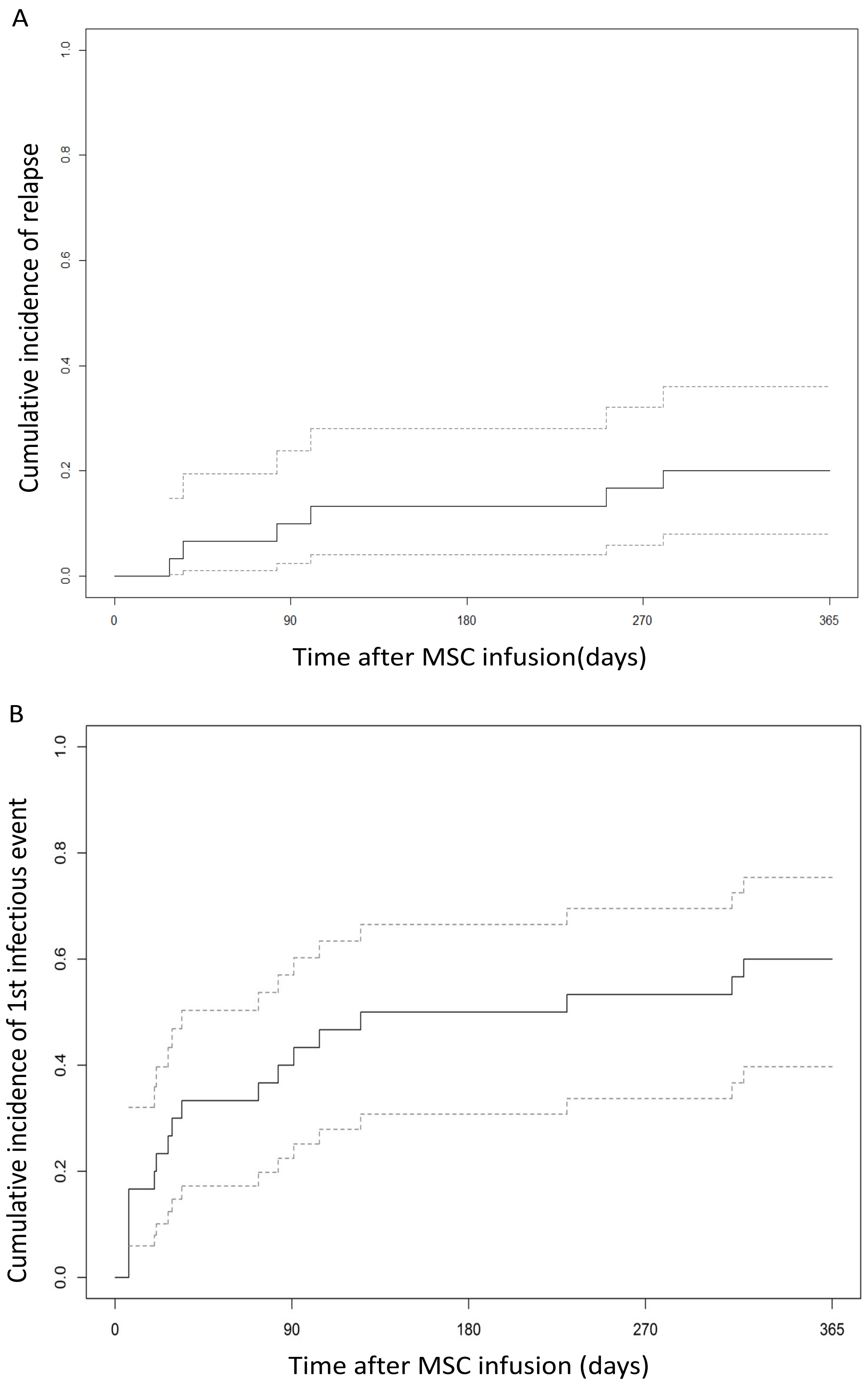

Supplement: Supplementary file 1 [file DataSheet_1.pdf]
